# Supplementary material for: Dissolving salt is not equivalent to applying a pressure on water
Source: Nat Commun. 2022 Feb 10;13:822. doi: 10.1038/s41467-022-28538-8 (PMC8831556; doi:10.1038/s41467-022-28538-8)
Supplement: Supplementary file 1 — Supplementary Information [file 41467_2022_28538_MOESM1_ESM.pdf]

Supplementary Information

Dissolving salt is not equivalent to applying a pressure on water

C. Zhang et al.

Supplementary Methods

Training of the deep neural network (DNN) model

An active machine learning approach called deep potential generator (DP-GEN)[1] was employed to construct an accurate and transferable DNN model suitable for both pure water and sodium chloride (NaCl) solutions, with different concentrations and under various pressures, at an *ab initio* level. The procedure is displayed in Supplementary Figure 1 and summarized as follows:

Step 0: Preparing initial training data. We extracted ∼4000 configurations of the NaCl solution (each configuration was modeled in a cell containing 1 NaCl ion pair and 62 water molecules) and ∼1000 configurations of pure water (each configuration was modeled in a cubic cell containing 64 water molecules) from previous *ab initio* molecular dynamics (AIMD) trajectories. The energy E, force $\mathbf{F}_i$ on each atom i, and the virial tensor $\boldsymbol{\Xi}$ of these configurations were calculated based on density functional theory (DFT)[2] as implemented in the Quantum ESPRESSO (QE)[3] code packages. The electron exchange-correlation was treated by the strongly constrained and appropriately normed (SCAN)[4] exchange-correlation functional approximation. Within the DFT calculations, the electron-nuclei interactions were treated by the pseudopotential approach. In particular, the Hamann-Schluter-Chiang-Vanderbilt (HSCV)[5,6] pseudopotentials were employed to the oxygen (O), hydrogen (H), and Cl atoms, while the Norm-Conserving Vanderbilt (ONCV)[7] pseudopotential was used for the Na atom. The 1s electron of H, $2s^2 2p^4$ electrons of O, $3s^2 3p^5$ electrons of Cl, and $2s^2 2p^6 3s^1$ electrons of Na were treated as valence electrons explicitly. In particular, the semicore electrons in Na are considered to be valence electrons in the above. A cutoff energy of 150 Ry for plane-wave expansions was adopted. The electronic ground state was considered to be converged based on a threshold of energy difference 1×10^{-6} Ry between consecutive self-consistent electronic iterations. Because of the large

supercells, the Brillouin zone integration was sampled at the Gamma point only. The obtained E, $\mathbf{F}_i$, and $\boldsymbol{\Xi}$ together with atomic positions were used as the initial training data.

Step 1: Training. Four DNN models were trained independently using the DeePMD-kit package[8] based on the same input training data, but different random seeds were used to initialize parameters of the neural networks. The deep potential method assumes that the energy of the system E can be written as the sum of atomic energies E_i[8,9]. The atomic energy E_i of atom i is a function of $\mathcal{R}_i$ which is the local environment of atom i in terms of the relative coordinates of its neighbors within a cutoff radius r_c. In this work, r_c was set to 6 Å. Firstly, $\mathcal{R}_i$ was encoded into the so-called feature matrix $\mathcal{D}_i$ to preserve the translational, rotational, and permutational symmetries[8,9]. Then, parameters of the DNN model that map $\mathcal{D}_i$ to E_i were optimized by minimizing the following loss function:

$$\mathcal{L}(p_\epsilon, p_f, p_\xi) = p_\epsilon \Delta\epsilon^2 + \frac{p_f}{3N}\sum_i |\Delta\mathbf{F}_i|^2 + \frac{p_\xi}{9} \parallel \Delta\boldsymbol{\xi} \parallel^2$$

where Δ denotes the difference between the training data and the current deep potential prediction, N is the number of atoms, $\epsilon \equiv E/N$, $\boldsymbol{\xi} = \Xi/N$. In the training process, the prefactors p_ϵ and p_ξ progressively increase from 0.02 to 8, while the prefactor p_f progressively decreases from 1000 to 1. The number of training steps was set to 1×10^6.

Step 2: Exploring. To explore the potential energy surface, we conducted molecular dynamics simulations of the pure water and NaCl solutions in the isobaric-isothermal (*NpT*) ensemble with a variety of initial configurations. NaCl solutions with the concentrations of NaCl : water mole ratios ranging from 1:83 to 1:10 were simulated at a pressure of 1 bar and temperatures of both 300 K and 333 K. The pure water system was simulated from 1 to 4000 bar at 333 K. In the simulations, one DNN model randomly selected from the four models produced in step 1 was

used to propagate the trajectory, and all the four models were employed to obtain the force acting on each atom at each timestep. The maximum standard deviation of the predicted atomic forces $\zeta = max_i\sqrt{\langle \| \mathbf{F}_i - \bar{\mathbf{F}}_i \|^2 \rangle}$ of each snapshot were calculated and used as an indicator for the convergence, where $\bar{\mathbf{F}}_i = \langle \mathbf{F}_i \rangle$ is the average force on atom i predicted by the four different DNN models.

Step 3: Labeling. The snapshots that have $\zeta > 0.15$ eV/Å were extracted. The E, $\mathbf{F}_i$, and $\mathbf{\Xi}$ of these snapshots were calculated using DFT with the same parameters as step 0 and then added to the training data set.

The loop from step 1 to step 3 was repeated until the configurations that have $\zeta > 0.15$ eV/Å account for less than 0.005 % of the total configurations in the exploration of the potential energy surface.

Deep potential molecular dynamics (DPMD) simulations

The converged DNN model was used to conduct DPMD production runs of both pure water and NaCl solutions within the *NpT* ensemble using LAMMPS[10] in conjunction with the DeePMD-kit package[8]. Pure water was simulated at 333 K and five different pressures (1, 1000, 2000, 3000, 3600 bar) modeled by a periodic cubic cell containing 512 water molecules. NaCl solutions were simulated at 333 K and 1 bar with eight different concentrations. The elevated simulation temperature by 35 K with respect to the experimental temperature of 298 K[11,12] is to compensate for the 35 K underestimation of the melting temperature of ice by the SCAN functional[13], which is mainly due to the deficiency of the SCAN functional that slightly overestimates the strength of hydrogen bonds. The numbers of NaCl ion pairs and water molecules contained in the periodic

cubic cell of DPMD simulations at different NaCl concentrations are listed in Supplementary Table 1. The concentrations of 1:83, 1:40, 1:17, 1:10 have been used in the neutron diffraction experiments[11,12] as well, therefore the corresponding theoretical predictions can be directly compared with experimental measurements. For each concentration of NaCl solution and each pressure for pure water, the DPMD simulation was carried out for 2 ns, in which the first 50 ps of the trajectory is discarded for equilibrium in the following analysis. The integration time step was set to 0.5 fs.

Validation of the DPMD simulations

Comparison with DFT

We uniformly extracted 180 configurations from the DPMD production-run trajectories. The energy and atomic forces of these configurations were calculated using DFT with the same parameters as that used in the preparation of the DNN training data. The energy and atomic forces predicted by DFT were compared with those predicted by the DNN model in Supplementary Figure 2 The DNN model reproduces well the DFT energy and atomic forces. The root-mean-squared errors of the energy and atomic force predicted by the DNN model with respect to DFT were 2.57×10^{-4} eV/atom and 7.24×10^{-2} eV/Å, respectively, indicating that the typical DPMD training accuracy[14] has been achieved.

We also compared the radial distribution functions (RDFs) predicted by the DPMD simulations with that of the AIMD results. For pure water, we compare the DPMD result with a previous AIMD simulation of pure water. As shown in Supplementary Figure 3a, b, the O-O and O-H RDFs ($g_{\mathrm{OO}}(r)$ and $g_{\mathrm{OH}}(r)$) predicted by the DPMD simulation agree well with the AIMD

results, indicating that our DPMD simulation of pure water can well reproduce the AIMD results[15]. For the NaCl solution, we carried out a 100 ps AIMD simulation at 1 bar and 300 K using a cubic cell containing one NaCl ion pair and 62 water molecules. The canonical (NVT) ensemble rather than the NpT ensemble was used because the absence of Pulay forces in the NVT simulation allows us to adopt a much lower energy cutoff of 85 Ry, which largely reduced the AIMD computational cost. We also conducted a 2 ns DPMD simulation in the NVT ensemble using the same simulation cell and thermodynamic condition. The $g_{\mathrm{OO}}(r)$, $g_{\mathrm{OH}}(r)$, $g_{\mathrm{ONa}}(r)$, and $g_{\mathrm{OCl}}(r)$ in Supplementary Figure 3c-f show that our DPMD simulation of the NaCl solution can well reproduce the AIMD results. It needs to be noted that the system presented in Supplementary Figure 3c-f only has one NaCl ion pair but 62 water molecules, so the convergence of $g_{\mathrm{ONa}}(r)$, and $g_{\mathrm{OCl}}(r)$ demands much longer simulation time than that of $g_{\mathrm{OO}}(r)$ and $g_{\mathrm{OH}}(r)$. Therefore, the $g_{\mathrm{ONa}}(r)$, and $g_{\mathrm{OCl}}(r)$ in Figs. S3e, f, predicted by the 100 ps AIMD simulation have large fluctuations. The computational efficiency of the DPMD simulation enables us to get better statistics.

The effect of long-range Coulombic interactions

As described in step 1 of the DNN training process, we have explicitly treated the atomic interactions within a radial cutoff distance of 6 Å. The interactions beyond the above range were neglected. In this section, we numerically prove that the RDFs predicted by the above cutoff distance of 6 Å are well converged. We show that further long-range interactions are not necessary for the calculation of RDFs because the long-range electrostatics are significantly screened in the condensed phase.

Firstly, the DFT energies and atomic forces in Supplementary Figure 2 were calculated using QE with cells larger than 24×24×24 Å^3, where the atomic interactions in the whole system were represented explicitly. The DNN atomic energies and forces, on the other hand, only treated the atomic interactions within 6 Å explicitly. However, the DNN results agree well with the DFT results, indicating that the long-range interactions only have a very small effect on the systems studied in this work. Secondly, the role of long-range interactions in the DPMD simulation has been explored in Ref. [16] which found that the neglect of explicit long-range interactions can induce errors in representing cluster and vapor phase properties but will not affect the condensed phase properties.

In this work, we applied the recently developed deep potential model with long-range correction (DPLR)[17] to check our main conclusion in the presence of the long-range electrostatic interaction. In DPLR simulations, the short-range contribution is treated as in the standard DP model, the long-range Coulombic interactions are implemented with the particle-particle particle-mesh (PPPM) solver[18] with spherical Gaussian charges located at the Wannier centroid.

The RDFs of the pure water and the NaCl solution predicted by the DPLR simulations are presented in Supplementary Figure 4 together with the original DP results for comparison. The agreement between the DP and DPLR results indicates that the long-range interactions have a negligible effect on the RDFs. Therefore, the explicit long-range representations beyond 6 Å are not necessary for the analyses in this work. However, for more delicate effects, such as the long-range water-water orientational correlations[19,20], the explicit treatment of the long-range Coulombic interactions are important.

Finite-size effects

The determination of the structure factor from simulations can suffer from finite-size effects, especially at low Q. To check the convergence of the structure factor, we modeled pure water with periodic cells containing 64, 512, and 4096 water molecules, respectively. As shown in Supplementary Figure 5, the simulation cells used in this work ($\sim$ 500 water molecules) is adequate for obtaining converged structure factors.

<u>The nuclear quantum effects on the molecular structure of NaCl solutions</u>

To explore the nuclear quantum effects on the structure of NaCl solutions, we conducted path-integral DPMD (PI-DPMD) simulations on NaCl solutions within the NpT ensemble by using the i-PI code[21] in connection with the DeePMD-kit package[8]. The nuclear degrees of freedom were sampled using eight beads with a colored-noise generalized Langevin equation thermostat[22,23] to accelerate the convergence of the quantum distribution. In order to enable a direct comparison with our classical simulations in the main text of the paper, the quantum simulations were also performed at 1 bar and 333 K. Limited by the significant computational cost of path-integral simulations, four concentrations of eight total concentrations were chosen to be studied. Each concentration was simulated for 120 ps with the first 20 ps discarded for equilibrium.

As shown in Supplementary Figure 6, nuclear quantum effects have a negligible effect on the structure factor $S_{\mathrm{XX}}(Q)$. This is because $S_{\mathrm{XX}}(Q)$ is mostly determined by the correlation between oxygen atoms; while nuclear quantum effects only have a more significant impact on the atoms with a small mass, for example, the hydrogen atom. In this work, we focus on the pressure-like effect in solutions which mainly manifest in the correlation between oxygen atoms. Therefore, the nuclear quantum effects are not included in the main manuscript.

The excluded volume correction

To explore the water structure beyond ionic first solvation shells, we excluded the water molecules located in ionic first solvation shells and calculated the O-O RDFs of the remaining free water (FW), which created some voids in the system. The voids have an “excluded volume" effect[11,24] on the RDF. This is a topological effect which results in higher intensity in the low r range for the RDF calculated with voids ($g_{void}(r)$) in comparison to that of the intact system ($g_{intact}(r)$). The physical origin is that, in the system with voids, the local density seen from a remaining atom is normally higher than the average number density of the system[11,24]. To compare the RDF of solutions calculated with voids with the RDF obtained from continuous pure water, the excluded volume effect needs to be eliminated. According to Ref. [24], the $g_{void}(r)$ can be corrected by dividing a “uniform atom distribution ($g_u(r)$)". That is to say, the corrected RDF can be expressed as $g_{void}^{c}(r) = g_{void}(r)/g_u(r)$.

For systems with non-overlap spherical voids, the expression of $g_u(r)$ has been deduced in Ref. [11,24]. However, the overlap of ionic first solvation shells is ubiquitous in NaCl solutions, especially at high concentrations. Thus, the expression of $g_u(r)$ in NaCl solutions is unknown. However, we can use homogeneous systems to obtain the $g_u(r)$ of NaCl solutions because the excluded volume effect is primarily a topological effect, and $g_u(r)$ mainly depends on the relative volume excluded from the simulation box rather than the atomic interactions[11,24]. We can consider the case of three different systems: pure water simulated using our DNN model, liquid Argon (Ar) simulated using a Lennard-Jones potential, and ideal gas which is not subject to any atomic interactions. Obviously, these three systems have completely different types of atomic interactions, which are also shown by their different RDFs ($g_{intact}(r)$ in Supplementary Figure 7). The

$g_{void}(r)$ of these systems can be calculated directly after excluding some voids in these systems. The purpose of the excluded volume correction is to eliminate the topological effects of the voids, which is equivalent to filling the voids such that the system is restored to a homogeneous system without voids. Therefore, the $g_{void}^{c}(r)$ should equal to $g_{intact}(r)$, and $g_u(r)$ of these homogeneous systems can be calculated as $g_u(r) = g_{void}(r)/g_{void}^{c}(r) = g_{void}(r)/g_{intact}(r)$. As shown in Supplementary Figure 7, when we exclude the same voids from each of the 3 systems, all of which have the same cell size, the resultant $g_u(r)$ for all 3 systems are equivalent, even though the atomic interactions of these systems are completely different. Therefore, the above $g_u(r)$ can be used for NaCl solutions.

In practice, for a specific NaCl solute concentration, the average cell size was calculated from the *NpT* DPMD trajectory. We then ran molecular dynamics simulations of an ideal gas using the same cell size. We then excluded the same voids in the ideal gas as that in the NaCl solution (For each timestep, all the voids in NaCl solutions were recorded. At each corresponding timestep of the trajectory of the ideal gas, the atoms located in the voids are excluded.) and calculated the $g_u(r)$. The $g_u(r)$ for NaCl solutions with low (1:83) and high (1:17) concentrations calculated in this way are displayed in Supplementary Figure 7a, b, respectively. The $g_{\mathrm{OO}}^{\mathrm{FW}}(r)$ of the NaCl solution were corrected by dividing the above $g_u(r)$. The $g_{\mathrm{OO}}^{\mathrm{FW}}(r)$ before correction were displayed in Supplementary Figure 8, while the $g_{\mathrm{OO}}^{\mathrm{FW}}(r)$ after correction were shown in Supplementary Figure 3d in the main manuscript.

Definition of hydrogen bonds

The hydrogen bonds between two water molecules are defined when the O-O distance is shorter than 3.5 Å and the O-O-H angle is smaller than 30°[25]. The hydrogen bonds between a water molecule and a Cl^{-} ion are defined when the Cl-O distance is shorter than 3.9 Å and the Cl-O-H angle is smaller than 30°[26].

Supplementary Discussion

The densities of water and NaCl solutions

The densities of pure water and NaCl solutions obtained from the DPMD simulations in the *NpT* ensemble are presented in Supplementary Figure 9 together with experimental results for comparison. The differences between the densities predicted by DPMD and the experimental values are smaller than 5%. Moreover, the varying trends of the density of pure water with pressure and NaCl solutions with concentration predicted by DPMD simulations agree well with experimental results. The densities obtained from DPMD simulations are slightly larger than the experimental results, which is consistent with the fact that the SCAN functional is known to overestimate the density of water[15]. The overestimated density of liquid water is mainly attributed to the inherit self-interaction error in the SCAN meta-GGA functional approximations. Because of the self-interaction error, the electron applies an unphysical self-interaction on itself which results in the delocalization error and overestimated hydrogen-bond strength[27]. The overestimated hydrogen-bond strength in turn brings the water molecules slightly too close to each other, which also overestimated the densities of water as well as the aqueous solutions. At the same time, the uncertainty in the predicted density of water and solutions can also arise from the neglect of the nonlocal electron correlation effect particularly at long-range of the hydrogen-bond network.

Composite partial structure factors

Apart from the composite partial structure factors $S_{\mathrm{XX}}(Q)$ presented in the main manuscript, the other two types of composite partial structure factors $S_{\mathrm{XH}}(Q)$ and $S_{\mathrm{HH}}(Q)$ can also be obtained from the neutron diffraction experiment[12]. $S_{\mathrm{XH}}(Q)$ contains the correlations of H

atoms with other types of atoms and can be calculated by $S_{\mathrm{XH}}(Q) = w_{\mathrm{OH}}S_{\mathrm{OH}}(Q) + w_{\mathrm{NaH}}S_{\mathrm{NaH}}(Q) + w_{\mathrm{ClH}}S_{\mathrm{ClH}}(Q)$, where $S_{\alpha\beta}(Q)$ is the partial structure factor between atomic types α and β (α, $\beta \in$ O, H, Na, Cl), $w_{\alpha\beta}$ is the weight of $S_{\alpha\beta}$'s contribution to $S_{\mathrm{XH}}(Q)$. $S_{\mathrm{HH}}(Q)$ is the correlation between H atoms in reciprocal space. The $S_{\mathrm{XH}}(Q)$ and $S_{\mathrm{HH}}(Q)$ of NaCl solutions obtained from the DPMD theoretical calculations are presented in Supplementary Figure 10 together with experimental results[12]. One can find that the DPMD simulations reproduced well the experimental $S_{\mathrm{XH}}(Q)$ and $S_{\mathrm{HH}}(Q)$.

Oxygen-ion RDFs

The O-Na and O-Cl RDFs, $g_{\mathrm{ONa}}(r)$ and $g_{\mathrm{OCl}}(r)$, in Supplementary Figure 11 show the solvation structure of the Na^+ and Cl^- ions. The $g_{\mathrm{ONa}}(r)$ has a more pronounced first peak than $g_{\mathrm{OCl}}(r)$, indicating that the Na^+ ion has a more rigid first solvation shell than the Cl^- ion. This is because the smaller ionic radius of Na^+ than Cl^- enables the Na^+ ion to bond water molecules more tightly in its first solvation shell. The radius of the first solvation shell of Na^+/Cl^- was defined as the position of the first minimum of the $g_{\mathrm{ONa}}(r)$/ $g_{\mathrm{OCl}}(r)$. Following this definition, when the concentration is increased from 1:83 to 1:10, the radius of the first solvation shells of Na^+ changes from 3.22 to 3.25 Å, while the radius of the first solvation shells of Cl^- changes from 3.84 to 4.07 Å.

Ion-ion RDFs and ion pairs

The ion-ion RDFs, $g_{\mathrm{NaCl}}(r)$, $g_{\mathrm{NaNa}}(r)$, and, $g_{\mathrm{ClCl}}(r)$ of NaCl solutions of different concentrations are displayed in Supplementary Figure 12. By integrating $g_{\mathrm{NaCl}}(r)$ from 0 to the

first minima (3.55 Å), we found that the number of Na-Cl contact ion pairs increases from 0.036 to 0.378, with the concentration increasing from 1:83 to 1:10. The first peak of $g_{\mathrm{NaCl}}(r)$ is located at 2.8 Å, which is smaller than the radius of the first solvation shells of both Na^{+} and Cl^{-} ions. Therefore, the overlap of hydration shells of ions is ubiquitous in NaCl solutions.

Coordination numbers

Figure S13 displays the distribution of the coordination numbers of the O atoms in the solvation shells of the Na^{+} and Cl^{-} ions. As shown in Supplementary Figure 13, most Na^{+} ions are five- or six- coordinated, while the coordination number of the Cl^{-} ion has a broad distribution from five to ten.

Analysis of hydrogen-bond network in NaCl solutions

We also conducted more detailed analyses on the hydrogen bond network using the joint probability of the proton transfer coordinate ν and the OH· · ·O angle θ. The increase of external pressure (Supplementary Figure 14b) and the addition of salt (Supplementary Figure 14d) both result in a broader distribution of ν and θ. However, after the ionic FSSs were excluded (Supplementary Figure 14c), the FW distribution in NaCl solution largely recovered that of pure water, which indicates that dissolving salt is not equivalent to applying a pressure on water.

Potassium chloride (KCl) and sodium bromide (NaBr) solutions

In order to elucidate the nature of the pressure-like effect of other salts in the Hofmeister series, we studied KCl and NaBr solutions systematically using the same method as that applied

for the NaCl solutions. As shown in Supplementary Figure 16a, the theoretical structure factor $S_{\mathrm{XX}}(Q)$ of KCl agrees well with the experimental results[11], which further validates the theoretical approach used in this work. The $S_{\mathrm{XX}}(Q)$ of NaBr in Supplementary Figure 16b is not compared with experiment due to the lack of available experimental results.

In the reciprocal space, the $S_{\mathrm{XX}}(Q)$ of both the KCl and NaBr solutions show an increasing first peak and diminishing second peak when salt concentration increases. In the real space, the $g_{\mathrm{OO}}(r)$ of both solutions show that the population of water molecules located in the interstitial region between the first and second peaks increases with increasing salt concentration, as displayed in the upper panel of Supplementary Figure 17. Concurrently, the second peak of the $g_{\mathrm{OO}}(r)$ collapses with increasing salt concentrations. The above changes in $S_{\mathrm{XX}}(Q)$ and $g_{\mathrm{OO}}(r)$ of KCl and NaBr solutions are consistent with that of NaCl solutions with increasing concentration and pure water with increasing external pressure.

When ionic FSSs are excluded, the $g_{\mathrm{OO}}^{\mathrm{FW}}(r)$ of both the KCl and NaBr solutions largely recover the $g_{\mathrm{oo}}(r)$ of pure water, as displayed in the lower panel of Supplementary Figure 17. Congruent with the results shown for the NaCl solutions, the pressure-like effects in KCl and NaBr solutions also originate from changes in water topology caused by the intrusion of ionic FSSs. Beyond ionic FSSs, ions do not have significant effects on the water structure.

Water-water orientational correlations in NaCl solutions

In principle, the H-bond network in liquid water can be represented by the correlation functions between any two water molecules $h(1,2) = h(\mathbf{r}, \boldsymbol{\mu}_1, \boldsymbol{\mu}_2)$. In the above, the indices $1,2$ represent any two water molecules separated by distance vector $\mathbf{r}$ in the models of liquid water or aqueous solutions, which carry out an electric dipole moment denoted by vectors $\boldsymbol{\mu}_1, \boldsymbol{\mu}_2$

respectively. This correlation function $h(1,2)$ can be further rigorously expanded systematically in terms of the rotational invariants by following the definitions in Refs. [28,29] as:

$$h(1,2) = h^{000}(r) + h^{110}(r)\phi^{110}(1,2) + h^{112}(r)\phi^{112}(1,2) + \cdots \quad (1),$$

where $\phi^{l_1 l_2 l}$ form an orthogonal basis for the angular dependence of $h(1,2)$, with

$$\phi^{110}(1,2) = \hat{\boldsymbol{\mu}}_1 \cdot \hat{\boldsymbol{\mu}}_2 \ (2), \ \ \phi^{112}(1,2) = 3(\hat{\boldsymbol{\mu}}_1 \cdot \hat{\mathbf{r}}) \cdot (\hat{\boldsymbol{\mu}}_2 \cdot \hat{\mathbf{r}}) - \hat{\boldsymbol{\mu}}_1 \cdot \hat{\boldsymbol{\mu}}_2 \ (3).$$

In Equation (1), the expansion coefficients (projectors) are determined by

$$h^{000}(r) =< h(1,2) >_{\boldsymbol{\mu}_1,\boldsymbol{\mu}_2} = g(r) - 1 \ \ (4),$$

$$h^{110}(r) = 3 < h(1,2)\phi^{110}(1,2) >_{\boldsymbol{\mu}_1,\boldsymbol{\mu}_2} \ (5)$$

$$h^{112}(r) = \frac{3}{2} < h(1,2)\phi^{112}(1,2) >_{\boldsymbol{\mu}_1,\boldsymbol{\mu}_2} \ (6).$$

In the current work, we have focused on elucidating the nature of the “pressure-like” effect measured by the structure factors in neutron scattering experiments. Structure factors are directly associated with the lowest-order correlation in Equation (1), i.e., the RDF $g(r) \equiv h^{000}(r) + 1$. The analyses on the RDFs indicate that, different from the pressure-like effect, ions have a relatively small effect on the water structure beyond FSSs. However, visible differences can still be seen between the FW structures in the NaCl solutions and neat water.

We noticed that some recent experimental and computational studies found that the solvated ions may be able to induce changes in terms of long-range orientation order in the water of the solvent[19,20,30,31]. These long-range orientational correlations are represented by higher-order terms h^{110} or h^{112}. Ion-induced modifications to the orientational correlation of water reported in the above literature is a relatively delicate effect, which shows up after multiplying a distance-weighted factor R^2. Therefore, the findings in this work (i.e., ionic have a small but visible effect)

do not go against the above literature[19,20,30,31]. To obtain a more comprehensive picture of ionic effects on the structure of water, we therefore also studied the water-water orientational correlations in NaCl solutions.

As discussed above, the ion-induced modification on the long-range orientational correlations is a delicate effect. The converged orientational correlations on the long-range ordering of the hydrogen bond network require a much larger simulation box and longer simulation time than what we presented in the main manuscript. Moreover, the long-range Coulombic interactions are important for precise descriptions of the orientational orders. Thus, we have conducted eight additional DPLR simulations on pure water and NaCl solutions with several concentrations using about 4000 water molecules. Each of which has been simulated for more than 15 ns and the metallic boundary condition is adopted. The details of the modeling of each NaCl concentration are listed in Supplementary Table 2.

As shown in Supplementary Figure 18a, we present the water-water orientational correlations $< cos\phi >$ (associated with h^{110} in equation (1) by a normalization factor) in NaCl solutions, where ϕ is the angle formed by the direction of the dipoles of any two water molecules in the system. It can be seen in Supplementary Figure 18a that $< cos\phi >$ deviates more and more from the neat water as the salt concentration increases. Even at the lowest concentration of 14 mM, the difference between NaCl solution and pure water is visible as displayed in Supplementary Figure 18b. The above results indicate that our SCAN-based DPLR simulation is in qualitative agreement with the finding in Ref. [19].

In order to further exclude the effect from ionic FSSs, in Supplementary Figure 18c, we presented the orientational correlations $< cos\phi >^{FW}$ of free water outside the FSSs. The comparison between Supplementary Figure 18c and Supplementary Figure 18a shows that most

of the modifications of the orientational correlation come from FSS water. After excluding FSS, $< cos\phi >^{FW}$ are very close to the $< cos\phi >$ of pure water. However, there are still visible differences between $< cos\phi >^{FW}$ and the $< cos\phi >$ of pure water even for NaCl solution with the lowest concentration studied in this work, as shown in Supplementary Figure 18d. The above findings are in accordance with the findings in the analyses of RDFs, i.e., ionic effects on water structure beyond FSSs are small but visible. Finally, it needs to note that the distance-weighted orientational correlations difference between dilute NaCl solutions and neat water presented in Supplementary Figure 18b, d have large fluctuations. In further studies, longer simulation time and larger simulation cells are expected to produce more converged results.

Supplementary Figures

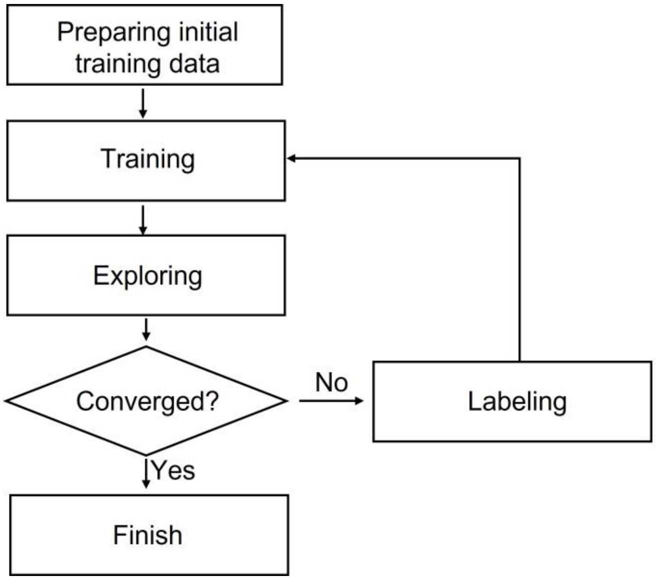

Supplementary Figure 1

The flow chart of the training procedure of the DNN model.

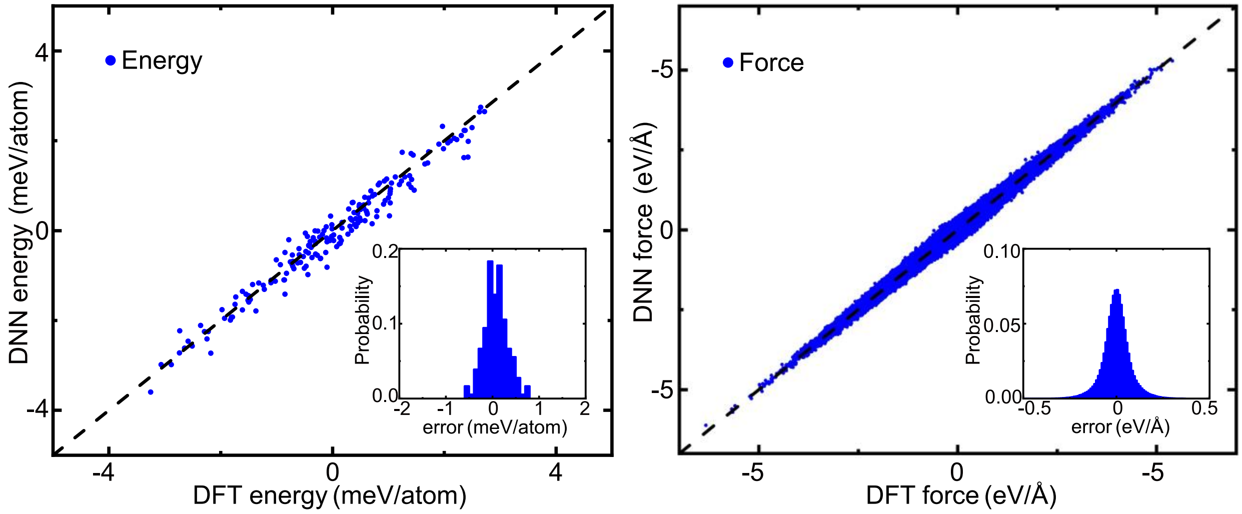

Supplementary Figure 2

Comparison of the energies and atomic forces predicted by the DNN model and SCAN DFT calculations. The average value of the energy of each system was shifted to zero for better visualization. The insets show the probability distributions of the differences between the energy and atomic force predicted by the DNN model with respect to those from SCAN DFT calculations.

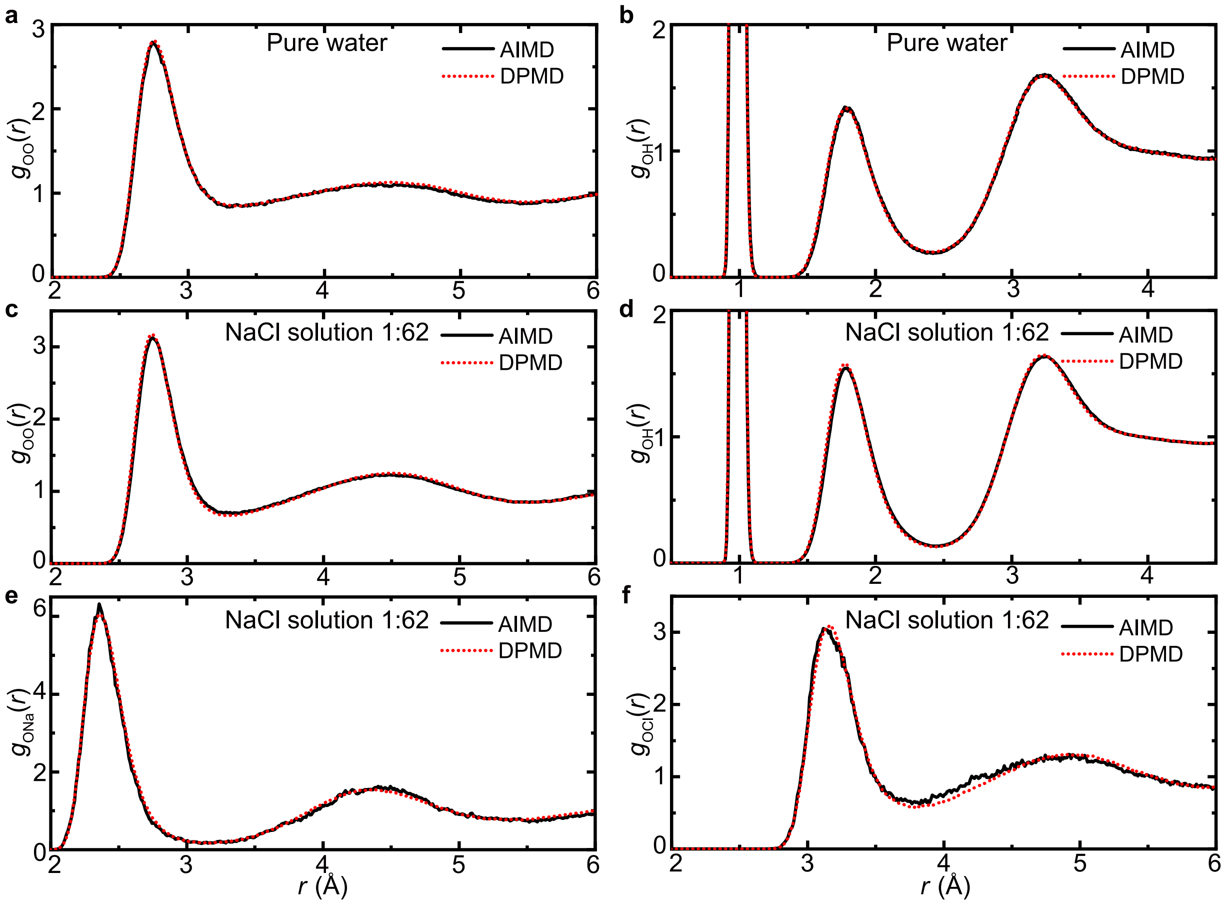

Supplementary Figure 3

a, $g_{\mathrm{OO}}(r)$ and **b,** $g_{\mathrm{OH}}(r)$ of pure water at 1 bar predicted by the DPMD simulation at 333 K and the AIMD simulation at 330 K [15]. Both DPMD and AIMD simulations were conducted in the *NpT* ensemble with a 64-molecule cubic cell. **c,** $g_{\mathrm{OO}}(r)$, **d,** $g_{\mathrm{OH}}(r)$, **e,** $g_{\mathrm{ONa}}(r)$, and **f,** $g_{\mathrm{OCl}}(r)$ of the NaCl solution at 1 bar and 300 K from DPMD and AIMD simulations. Both DPMD and AIMD simulations were conducted in the *NVT* ensemble with a simulation cell containing one NaCl ion pair and 62 water molecules.

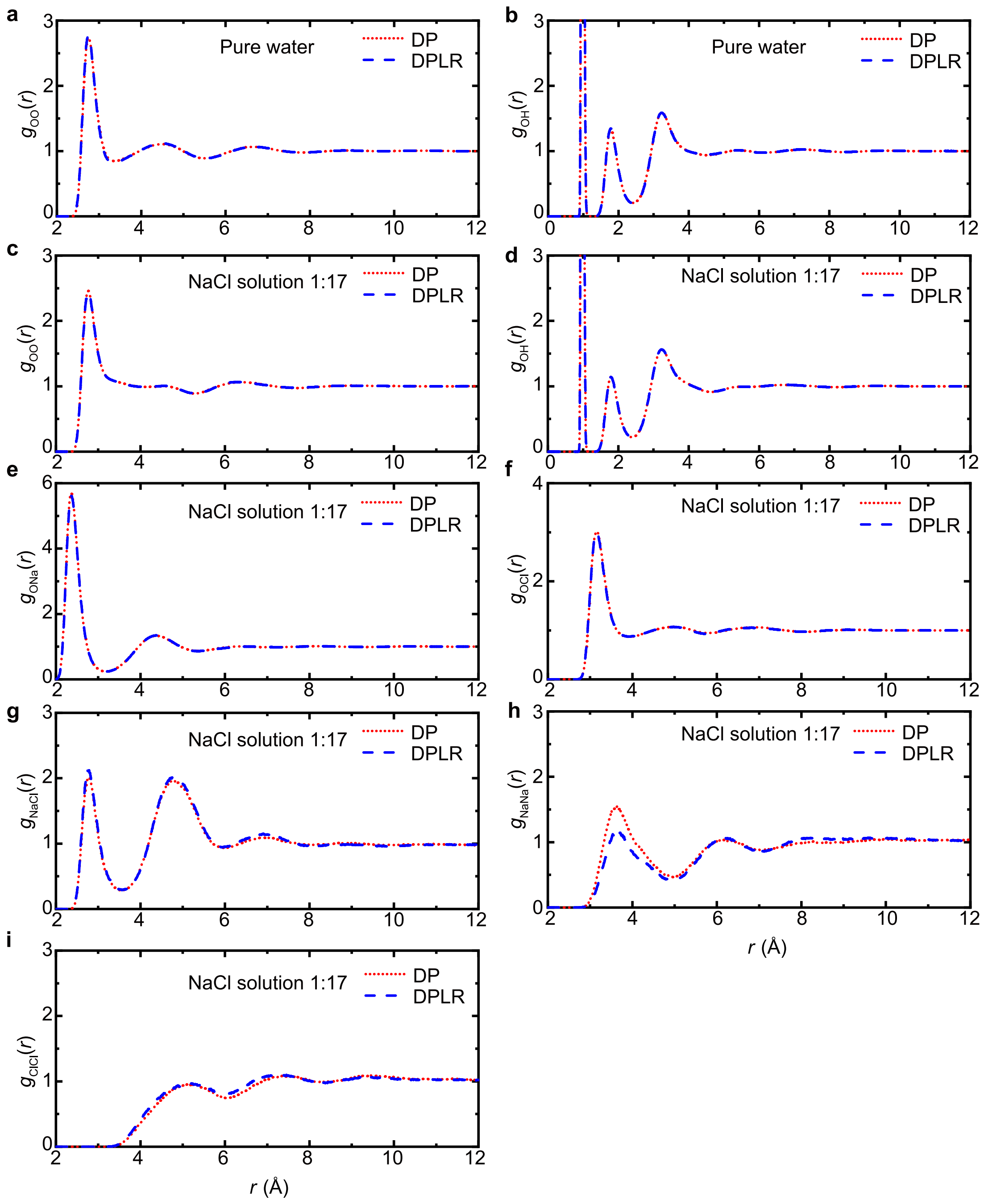

Supplementary Figure 4

a, $g_{\mathrm{OO}}(r)$ and **b,** $g_{\mathrm{OH}}(r)$ of pure water, **c,** $g_{\mathrm{OO}}(r)$, **d,** $g_{\mathrm{OH}}(r)$, **e,** $g_{\mathrm{ONa}}(r)$, **f,** $g_{\mathrm{OCl}}(r)$, **g,** $g_{\mathrm{NaCl}}(r)$, **h,** $g_{\mathrm{NaNa}}(r)$ and **i,** $g_{\mathrm{ClCl}}(r)$ of the NaCl solution with the concentration of 1:17 predicted by DP and DPLR simulations.

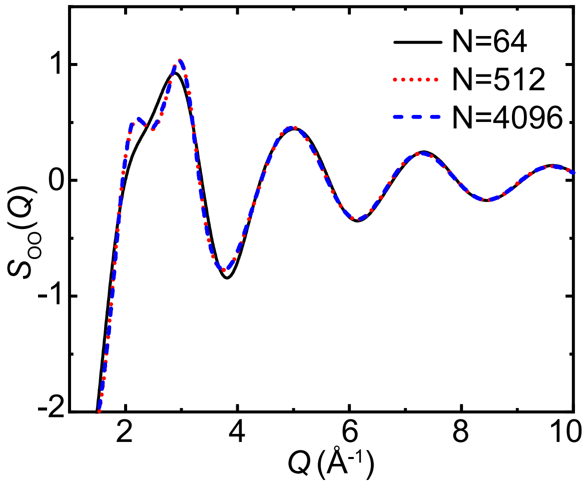

Supplementary Figure 5

$S_{OO}(Q)$ of pure water at 1 bar simulated using periodic cells containing 64, 512, and 4096 water molecules, respectively.

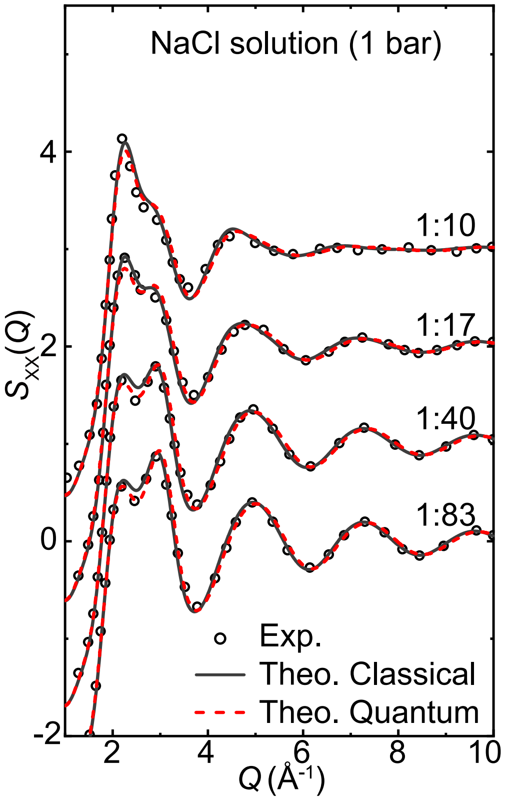

Supplementary Figure 6

Composite partial structure factors $S_{\mathrm{xx}}(Q)$ for NaCl aqueous solutions with salt : water mole ratios 1:83 to 1:10, at 1 bar from experiment [12], classical and quantum theoretical calculations. The structure factors were shifted vertically for visual clarity.

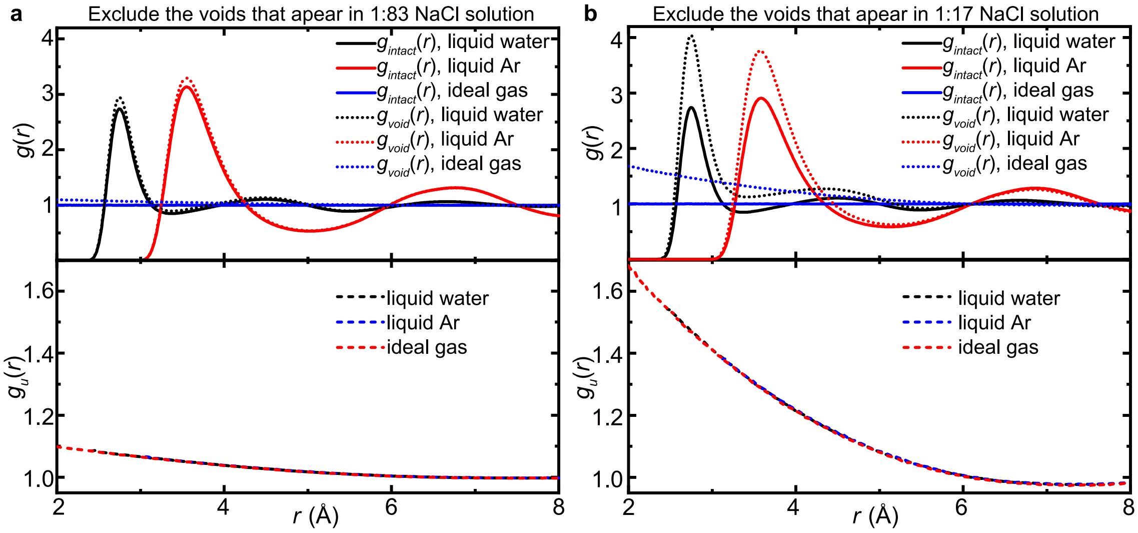

Supplementary Figure 7

$g_{intact}(r)$ and $g_{void}(r)$ of pure liquid water, liquid Ar, and ideal gas. The cell size and volumes excluded in the systems in **a,** and **b,** are equivalent to that of the 1:83 and 1:17 NaCl solutions, respectively. The $g_u(r)$ was calculated using $g_{void}(r)/g_u(r)$.

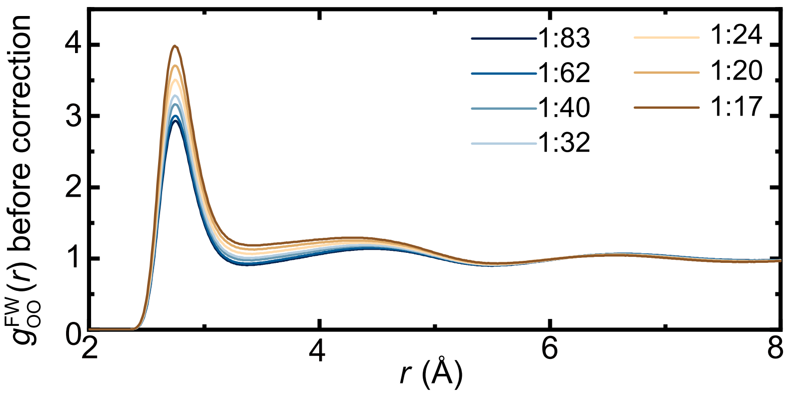

Supplementary Figure 8

$g_{OO}^{FW}(r)$ of NaCl solutions before the excluded volume correction.

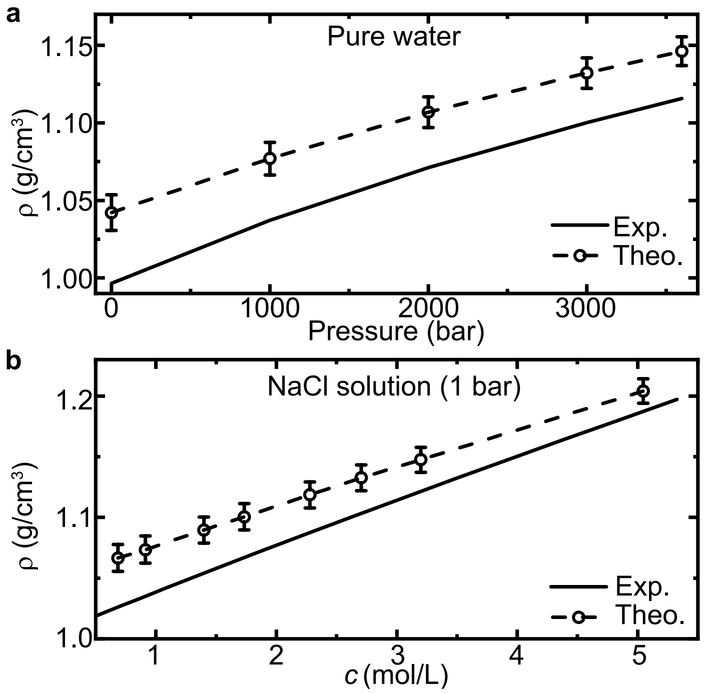

Supplementary Figure 9

a, Densities of pure water as a function of pressure from the theoretical DPMD calculations at 333 K and experiment at 300 K[32]. **b,** Densities of NaCl solutions at 1 bar as a function of solute concentration from theoretical DPMD calculation at 333 K and experiment at 293 K [33].

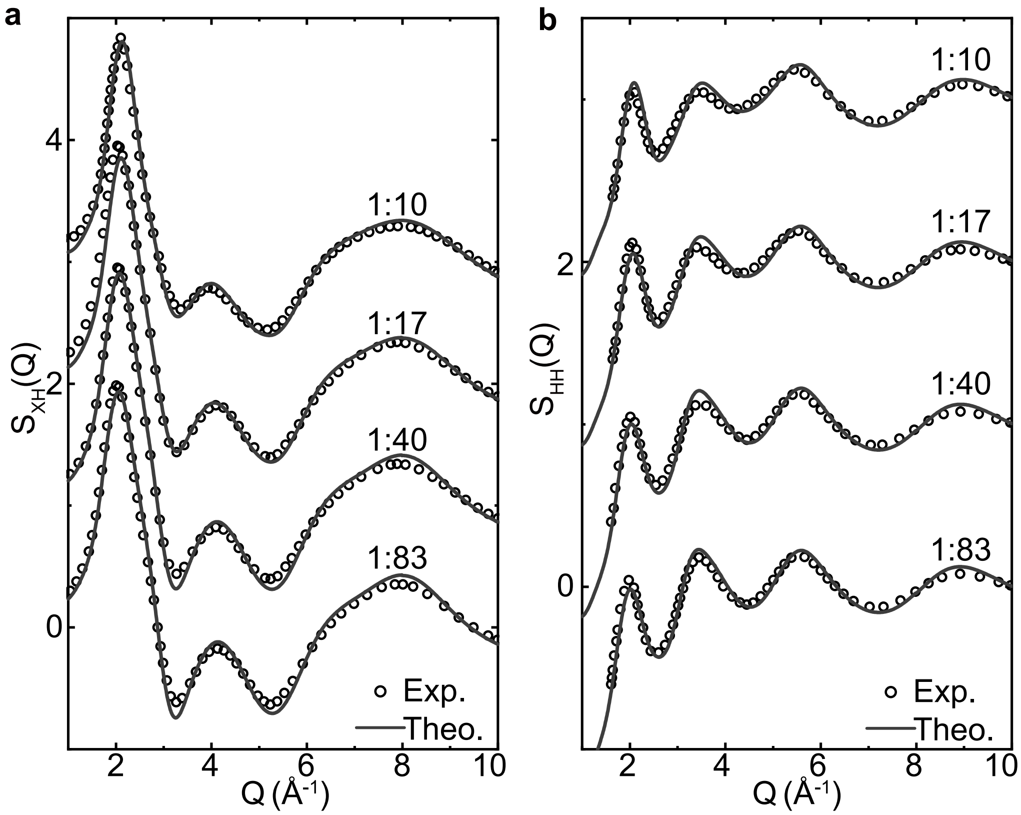

Supplementary Figure 10

a, $S_{\mathrm{XH}}(Q)$ and **b,** $S_{\mathrm{HH}}(Q)$ of NaCl solutions at 1 bar and different concentrations from experiment[12] and DPMD theoretical calculations. The structure factors were shifted vertically for visual clarity.

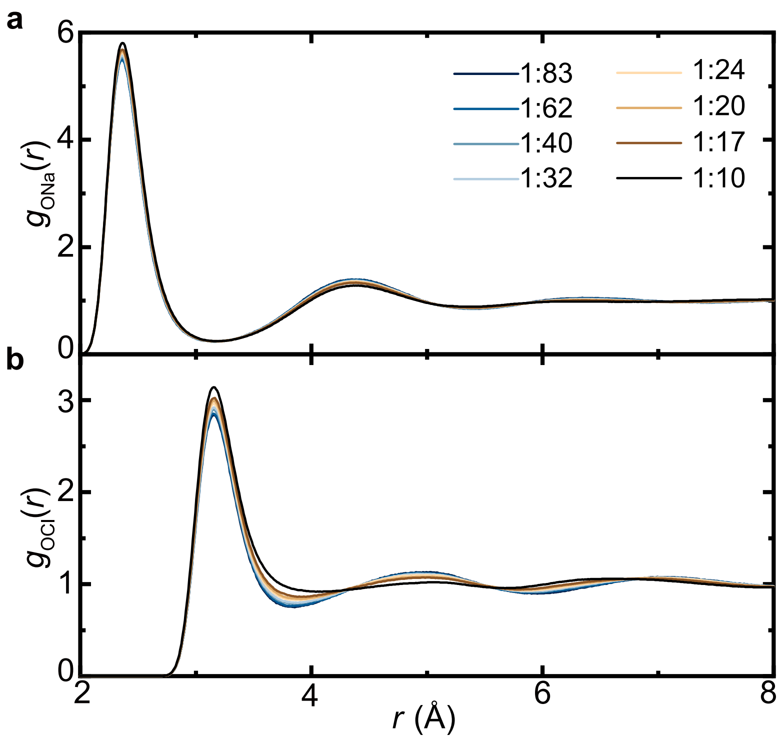

Supplementary Figure 11

a, $g_{\mathrm{ONa}}(r)$ and **b,** $g_{\mathrm{OCl}}(r)$ of NaCl solutions of different concentrations obtained from the DPMD simulations at 1 bar and 333 K.

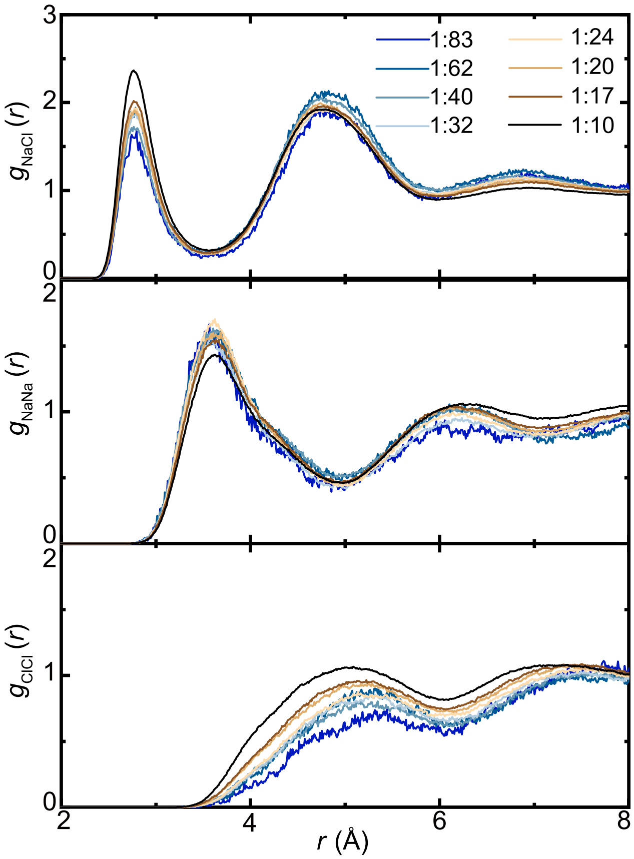

Supplementary Figure 12

Ion-ion RDFs, $g_{\mathrm{NaCl}}(r)$, $g_{\mathrm{NaNa}}(r)$, and, $g_{\mathrm{ClCl}}(r)$ of NaCl solutions of different concentrations obtained from the DPMD simulations at 1 bar and 333 K.

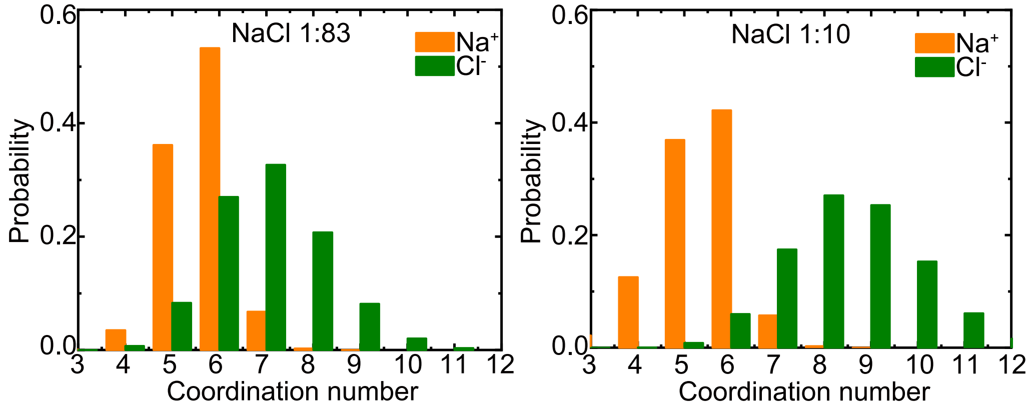

Supplementary Figure 13

Distribution of the coordination numbers of the O atoms in the solvation shell of the Na^+ and Cl^- ions at the concentration of 1:83 and 1:10, which shows the number of vertices of polyhedra formed by Na^+ and Cl^- ions with surrounding water molecules.

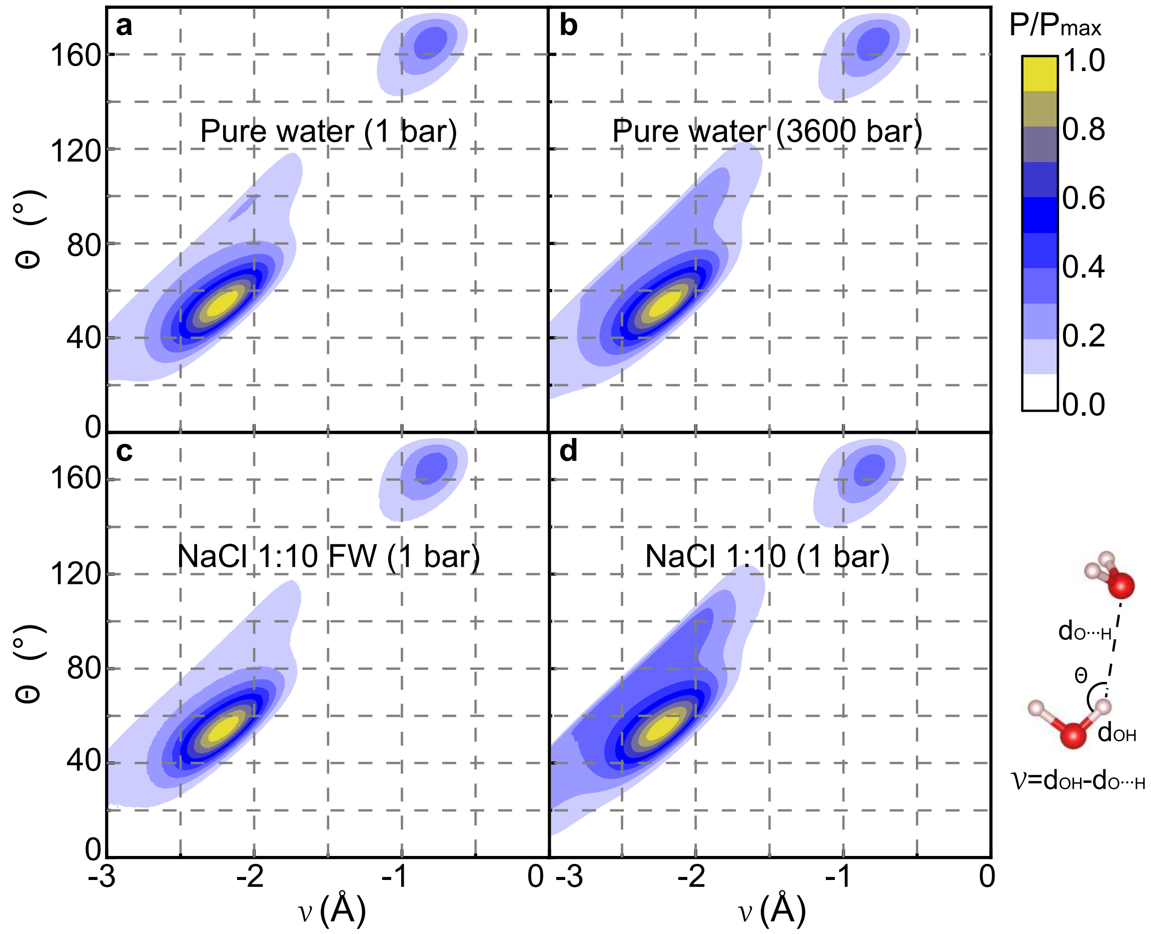

Supplementary Figure 14

Joint probability of hydrogen bonded geometries sampled from DPMD simulations of **a,** pure water, **b,** pure water at high pressure, **c,** 1:10 NaCl solution with only FW counted, and **d,** 1:10 NaCl solution with all water molecules counted as a function of the proton transfer coordinate ν and the OH$\cdot\cdot\cdot$O angle θ. The inset shows definitions of ν and θ.

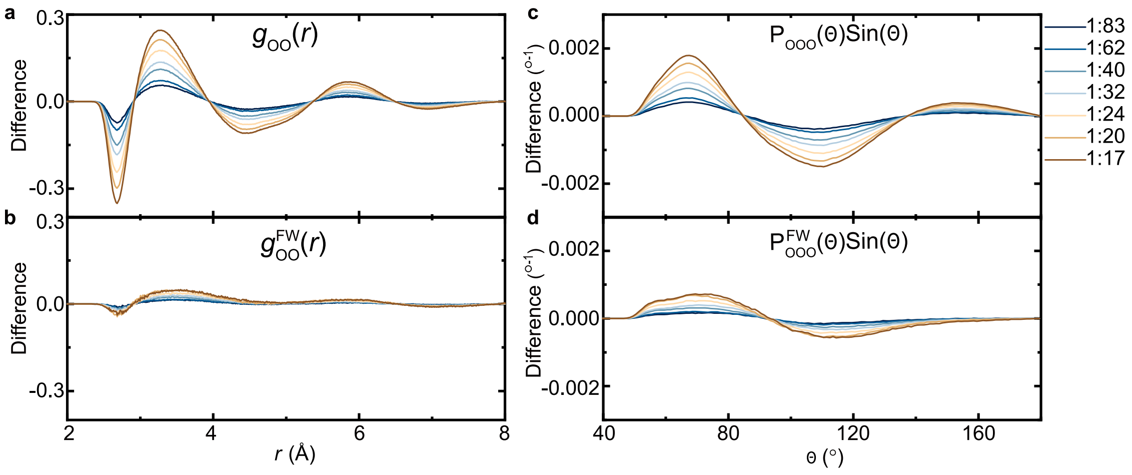

Supplementary Figure 15

NaCl – neat water (1bar) differences of **a,** $g_{OO}(r)$, **b,** $g_{oo}^{FW}(r)$, **c,** $P_{OOO}(\theta)$, and **d,** $P_{OOO}^{FW}(\theta)$.

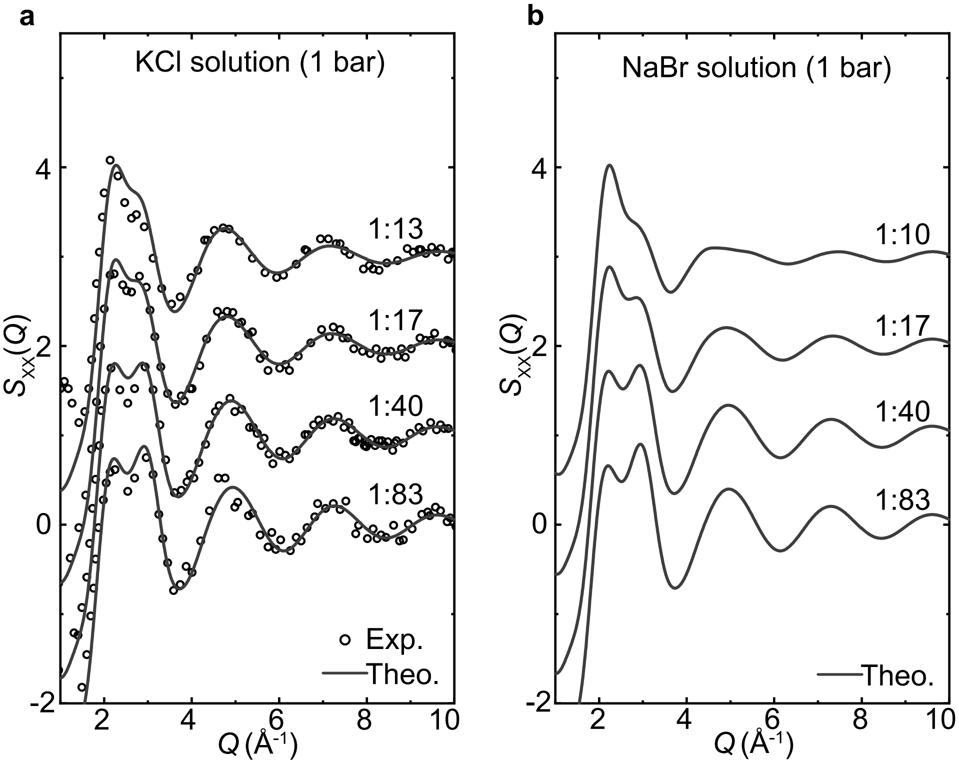

Supplementary Figure 16

a, Experimental[11] and theoretical $S_{\mathrm{XX}}(Q)$ for KCl solutions with salt : water mole ratios from 1:83 to 1:13, at 1 bar. **b,** Theoretical $S_{\mathrm{XX}}(Q)$ for NaBr solutions with salt : water mole ratios from 1:83 to 1:10, at 1 bar. All structure factors were shifted vertically for visual clarity.

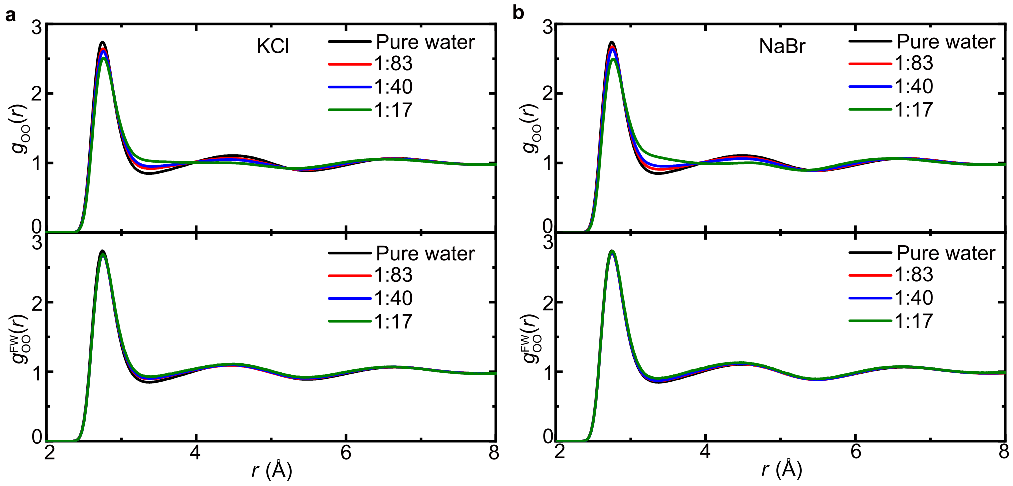

Supplementary Figure 17

a, $g_{oo}(r)$ and $g_{oo}^{FW}(r)$ for KCl solutions at various indicated concentrations in comparison with the $g_{oo}(r)$ of pure water. **b,** $g_{oo}(r)$ and $g_{oo}^{FW}(r)$ for NaBr solutions at various indicated concentrations in comparison with the $g_{oo}(r)$ of pure water.

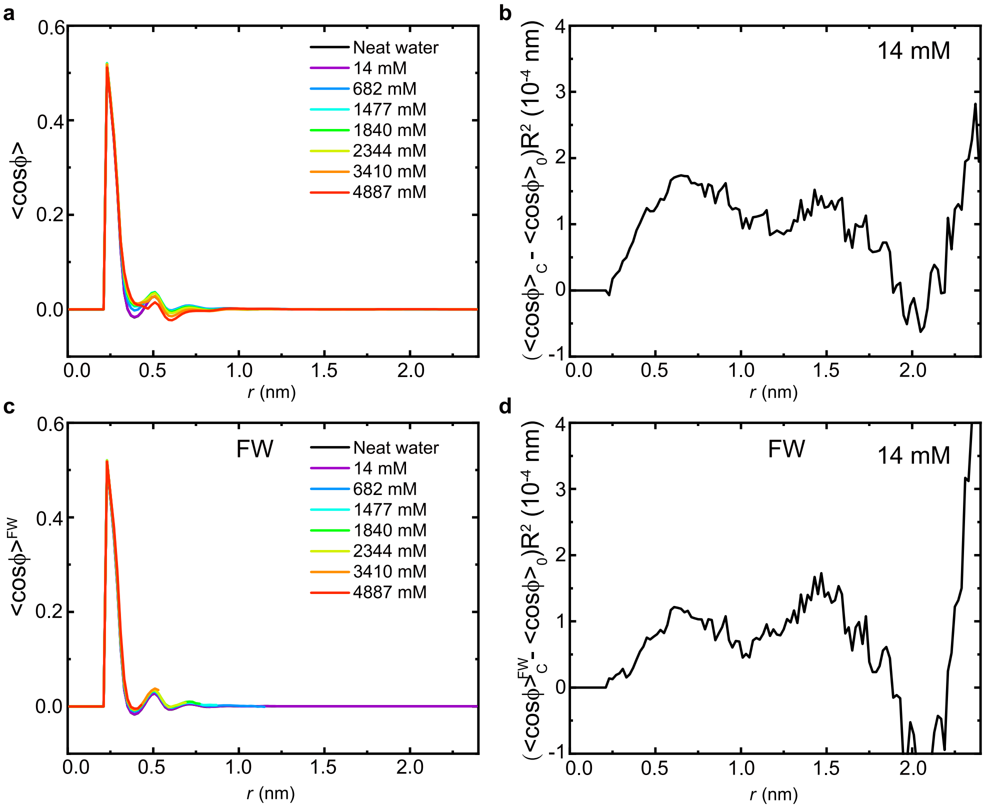

Supplementary Figure 18

SCAN DPLR simulation results of **a,** water orientational correlations of neat water and NaCl solutions with various indicated concentrations. **b,** ion-induced change in the orientational order for a 14 mM NaCl. **c,** orientational correlations of FW in NaCl solutions in comparison with the orientational correlations of neat water. **d,** ion-induced change in the FW orientational order for a 14 mM NaCl. All the simulations were conducted with the metal boundary condition. (In the calculation of $< cos\phi >^{FW}$, the angle ϕ between the central FW molecule and the surrounding FW molecule within a distance r is calculated. r is the distance between the central FW molecule and its nearest neighboring FSS molecule. Therefore, $< cos\phi >^{FW}$ truncations at a shorter distance with increasing salt concentrations.)

Supplementary Tables

N_{NaCl}: N_{H_2O}	c (mol/L)	N_{NaCl}	N_{H_2O}	P_{FSS} (%)
1:83	0.68	6	500	14.4
1:62	0.91	8	496	18.8
1:40	1.40	13	520	28.1
1:32	1.73	16	512	34.4
1:24	2.28	21	504	43.7
1:20	2.70	25	500	50.6
1:17	3.20	30	500	58.1
1:10	5.05	50	500	80.7

Supplementary Table 1

The number of NaCl ion pairs, N_{NaCl}, and water molecules, N_{H_2O}, contained in the periodic cubic cell of DPMD simulations at different NaCl concentrations c and the proportion of water molecules located in ionic first solvation shells P_{FSS}.

c (mM)	N_{NaCl}	N_{H_2O}
0	0	4056
14	1	4055
682	48	4000
1477	104	3920
1840	130	3898
2344	166	3855
3410	240	3720
4887	344	3560

Supplementary Table 2

The number of NaCl ion pairs, N_{NaCl}, and water molecules, $N_{\mathrm{H_2O}}$, contained in the periodic cubic cell of SCAN DPLR simulations at different NaCl concentrations c.

Supplementary References

1. Zhang, Y. *et al.* DP-GEN: A concurrent learning platform for the generation of reliable deep learning based potential energy models. *Comput. Phys. Commun.* **253,** 107206 (2020).
2. Hohenberg, P. & Kohn, W. Inhomogeneous Electron Gas. *Phys. Rev.* **136**, B864–B871 (1964).
3. Giannozzi, P. *et al.* Advanced capabilities for materials modelling with Quantum ESPRESSO. *J. Phys. Condens. Matter* **29**, 465901 (2017).
4. Sun, J., Ruzsinszky, A. & Perdew, J. P. Strongly Constrained and Appropriately Normed Semilocal Density Functional. *Phys. Rev. Lett.* **115**, 036402 (2015).
5. Hamann, D. R., Schlüter, M. & Chiang, C. Norm-Conserving Pseudopotentials. *Phys. Rev. Lett.* **43**, 1494–1497 (1979).
6. Vanderbilt, D. Optimally smooth norm-conserving pseudopotentials. *Phys. Rev. B* **32**, 8412–8415 (1985).
7. Hamann, D. R. Optimized norm-conserving Vanderbilt pseudopotentials. *Phys. Rev. B* **88**, 085117 (2013).
8. Wang, H., Zhang, L., Han, J. & E, W. DeePMD-kit: A deep learning package for many-body potential energy representation and molecular dynamics. *Comput. Phys. Commun.* **228**, 178–184 (2018).
9. Zhang, L. *et al.* End-to-end Symmetry Preserving Inter-atomic Potential Energy Model for Finite and Extended Systems. in *Advances in Neural Information Processing Systems 31* (eds. Bengio, S. et al.) 4436–4446 (Curran Associates, Inc., 2018).
10. Plimpton, S. Fast Parallel Algorithms for Short–Range Molecular Dynamics. *J. Comput. Phys.* **117(1),** 1-19 (1995).

11. Mancinelli, R., Botti, A., Bruni, F., Ricci, M. A. & Soper, A. K. Perturbation of water structure due to monovalent ions in solution. *Phys. Chem. Chem. Phys.* **9**, 2959 (2007).
12. Mancinelli, R., Botti, A., Bruni, F., Ricci, M. A. & Soper, A. K. Hydration of Sodium, Potassium, and Chloride Ions in Solution and the Concept of Structure Maker/Breaker. *J. Phys. Chem. B* **111**, 13570–13577 (2007).
13. Piaggi, P. M., Panagiotopoulos, A. Z., Debenedetti, P. G. & Car, R. Phase Equilibrium of Water with Hexagonal and Cubic Ice Using the SCAN Functional. *J. Chem. Theory Comput.* **17**, 3065–3077 (2021).
14. Zhang, L., Wang, H., Car, R. & E, W. Phase Diagram of a Deep Potential Water Model. *Phys. Rev. Lett.* **126**, 236001 (2021).
15. Chen, M. *et al.* Ab initio theory and modeling of water. *Proc. Natl. Acad. Sci.* **114**, 10846–10851 (2017).
16. Yue, S. *et al.* When do short-range atomistic machine-learning models fall short? *J. Chem. Phys.* **154**, 034111 (2021).
17. Zhang, L., Wang, H., Muniz, M. C., Panagiotopoulos, A. Z., & Car, R. A deep potential model with long-range electrostatic interactions. *arXiv preprint arXiv:*2112.13327 (2021).
18. Buneman, O. Computer Simulation Using Particles (R. W. Hockney and J. W. Eastwood). *SIAM Rev.* **25**, 2 (1983).
19. Chen, Y. *et al.* Electrolytes induce long-range orientational order and free energy changes in the H-bond network of bulk water. *Sci. Adv.* **2**, e1501891 (2016).
20. Borgis, D., Belloni, L. & Levesque, M. What Does Second-Harmonic Scattering Measure in Diluted Electrolytes? *J. Phys. Chem. Lett.* **9**, 3698–3702 (2018).

21. Ceriotti, M., More, J. & Manolopoulos, D. E. i-PI: A Python interface for ab initio path integral molecular dynamics simulations. *Comput. Phys. Commun.* **185**, 1019–1026 (2014).
22. Ceriotti, M., Bussi, G. & Parrinello, M. Nuclear Quantum Effects in Solids Using a Colored-Noise Thermostat. *Phys. Rev. Lett.* **103**, 030603 (2009).
23. Ceriotti, M. & Manolopoulos, D. E. Efficient First-Principles Calculation of the Quantum Kinetic Energy and Momentum Distribution of Nuclei. *Phys. Rev. Lett.* **109**, 100604 (2012).
24. Soper, A. K. The excluded volume effect in confined fluids and liquid mixtures. *J. Phys. Condens. Matter* **9**, 2399–2410 (1997).
25. Luzar, A. & Chandler, D. Hydrogen-bond kinetics in liquid water. *Nature* **379**, 55–57 (1996).
26. Wang, Q., Suzuki, K., Nagashima, U., Tachikawa, M. & Yan, S. Geometric isotope effects on small chloride ion water clusters with path integral molecular dynamics simulations. *Chem. Phys.* **426**, 38–47 (2013).
27. Zhang, C. *et al.* Modeling Liquid Water by Climbing up Jacob's Ladder in Density Functional Theory Facilitated by Using Deep Neural Network Potentials. *J. Phys. Chem. B* **125**, 11444–11456 (2021).
28. Gubbins, K. E. *Theory of molecular fluids*. (Oxford University Press, 1985).
29. Ballenegger, V. & Hansen, J.-P. Structure and dielectric properties of polar fluids with extended dipoles: results from numerical simulations. *Mol. Phys.* **102**, 599–609 (2004).
30. Duboisset, J. & Brevet, P.-F. Salt-induced Long-to-Short Range Orientational Transition in Water. *Phys. Rev. Lett.* **120**, 263001 (2018).

31. Wilkins, D. M., Manolopoulos, D. E., Roke, S. & Ceriotti, M. Communication: Mean-field theory of water-water correlations in electrolyte solutions. *J. Chem. Phys.* **146**, 181103 (2017).

32. Lemmon, E. W., McLinden, M. O. & Friend, D. G. Thermophysical properties of fluid systems. In *NIST Chemistry WebBook*, NIST Standard Reference Database Number 69, Eds. P.J. Linstrom and W.G. Mallard, National Institute of Standards and Technology, Gaithersburg MD, 20899, https://doi.org/10.18434/T4D303.

33. Haynes, W. M. *CRC Handbook of Chemistry and Physics*. (CRC Press, 2014).
